# Sociodemographic differences in linkage error: an examination of four large-scale datasets

**DOI:** 10.1186/s12913-018-3495-x

**Published:** 2018-09-03

**Authors:** Sean Randall, Adrian Brown, James Boyd, Rainer Schnell, Christian Borgs, Anna Ferrante

**Affiliations:** 1 Centre for Data Linkage, Curtin University, Perth, 6849 Western Australia; 2 German Record Linkage Center, University of Duisburg-Essen, D-47057 Duisburg, Germany

**Keywords:** Record linkage, Bias, Sociodemographic differences, Methodological research

## Abstract

**Background:**

Record linkage is an important tool for epidemiologists and health planners. Record linkage studies will generally contain some level of residual record linkage error, where individual records are either incorrectly marked as belonging to the same individual, or incorrectly marked as belonging to separate individuals. A key question is whether errors in linkage quality are distributed evenly throughout the population, or whether certain subgroups will exhibit higher rates of error. Previous investigations of this issue have typically compared linked and un-linked records, which can conflate bias caused by record linkage error, with bias caused by missing records (data capture errors).

**Methods:**

Four large administrative datasets were individually de-duplicated, with results compared to an available ‘gold-standard’ benchmark, allowing us to avoid methodological issues with comparing linked and un-linked records. Results were compared by gender, age, geographic remoteness (major cities, regional or remote) and socioeconomic status.

**Results:**

Results varied between datasets, and by sociodemographic characteristic. The most consistent findings were worse linkage quality for younger individuals (seen in all four datasets) and worse linkage quality for those living in remote areas (seen in three of four datasets). The linkage quality within sociodemographic categories varied between datasets, with the associations with linkage error reversed across different datasets due to quirks of the specific data collection mechanisms and data sharing practices.

**Conclusions:**

These results suggest caution should be taken both when linking younger individuals and those in remote areas, and when analysing linked data from these subgroups. Further research is required to determine the ramifications of worse linkage quality in these subpopulations on research outcomes.

**Electronic supplementary material:**

The online version of this article (10.1186/s12913-018-3495-x) contains supplementary material, which is available to authorized users.

## Background

Record linkage is a set of methodologies designed to bring together information relating to the same person from within or across datasets [1]. This technique is widely used for conducting longitudinal observational health research [2]. The process of record linkage typically involves the comparison of personally identifying information such as name, address and date of birth contained in these records.

Studies using linked data will generally contain linkage error. There are two types of record linkage errors; false positives, where two records are designated as belonging to the same individual when in truth they do not; and false negatives, where two records are designated as belonging to different individuals when in truth they belong to the same individual. Linkage error can occur due to both legitimate changes in a person’s particulars (i.e. change of address) or due to data fields being missing or in error (i.e. poor recording practices in the data collection in question). Researchers using linked data will generally not have the personally identifying information made available to them for privacy reasons [3]. This means they are unable to evaluate the accuracy or quality of the linkage directly and instead must rely on the quality provided by the organisation that performs the linkage.

A number of studies have illustrated how poor linkage quality can cause bias and distort results. A study of the effect of linkage methods (two deterministic strategies and one probabilistic strategy) on mortality rate estimates showed relative differences of up to 25% between the true estimate and that found through record linkage [4]. In a study on child neglect which applied an iterative linkage approach (deterministic followed by probabilistic) to reduce false positives, errors in linkage quality were shown to bias incidence proportions by up to 43% [5]. The incidence proportions were based on the number of children under the age of 6 years from the 2009 births to Alaska residents with at least one multi-source maltreatment report. In a recent study of men with and without HIV, the probabilistic linkage method applied (with estimated sensitivity and specificity of 88.4 and 99.7 respectively) led to a finding of a significantly lower rate of hospitalisation in HIV positive men as compared to the general population (0.46, 0.37–0.58), while improvements in linkage quality revealed the opposite finding; a significantly higher rate of hospitalisation in HIV positive men (1.45, 1.33–1.59) [6].

A key question is whether errors in linkage quality are distributed evenly across a study population. In other words, do the linked records of people from certain subgroups (for instance, people with lower socioeconomic status or individuals in particular ethnic groups) contain a greater proportion of errors? If a certain subgroup was found to have lower linkage quality, this would suggest research results might be systematically biased against this group.

There are a number of plausible reasons why linkage quality may vary between subgroups. Key causes of linkage error are changing or incorrect identifiers [7]. Given the common cultural norm of women changing their surname upon marriage, women may have a higher rate of linkage errors than men. Individuals who are more mobile (that is, change address often), such as younger adults, may also be harder to correctly link, resulting in more errors. Individuals from different ethnic groups may have their name information recorded poorly [8] (for instance due to difficulty spelling, or different transliterations), or may use name conventions different from Western standards (for instance, a very large proportion of Vietnamese women have the same middle name; *Thi*), which will make their data harder to link. Different ethnic groups may also display different rates of identifier reporting; for instance, in the United States, African American adults are less likely to report social security number, a highly identifying attribute, making their data harder to link [9]. Recording practices may differ between different hospitals, or different types of hospitals, which may service differing constituencies. Of importance to health researchers is knowing whether linkage errors differ between socioeconomic status and by geographic region (metropolitan compared to rural), as these are two key demographic factors known to influence health status [10]. Different health conditions may also be correlated with missing or invalid identifiers; for instance, medical conditions relating to newborns who may not have recorded first names [11]. A recent study of the existence of identifier error in administrative datasets showed the level of error to vary by age, sex and ethnicity [12], indicating the potential for differences in linkage quality across these attributes.

A number of studies have explored the relationship between linkage quality and sociodemographic factors, although typically as part of a wider study. A review by Bohensky et al. [13] found differences in age, sex, ethnicity, geography, socio-economic status and health, although there was little consistency between studies. In general, these studies of variations in linkage quality across or within populations have used the same research design. In nearly all cases, the study method involved the linkage of two datasets, where each dataset contained only one record per person. Each record in the first dataset was expected to be contained within the second dataset. Using this method, records in the first dataset could be divided into two categories; those that matched with a record in the second dataset and those that did not. These unmatched records were then compared against the matched records to determine whether there was any difference in the social and demographic characteristics (i.e. gender, age, socioeconomic status) of these groups [14–27].

Two key issues arise when using this research design to explore bias caused by poor linkage quality. Firstly, the approach focusses only on false negative errors (records which incorrectly did not find a match) and does not consider the issue of false positive errors, an equally important type of linkage error. By only reporting on one of the two error types, we cannot gain an accurate understanding of the relationship between linkage quality and sociodemographic factors.

A second and more fundamental problem in comparing sociodemographic subgroups using this methodology is that we often cannot distinguish whether an individual is unmatched due to linkage quality error, or due to the fact that these matched records do not exist. In many of these cases, differences in linkage bias arise from differences in subgroup data capture. Such differences can be expected to be highly dataset dependent. For example, in a recent US study linking a clinical surgical registry to US Medicare inpatient claims, the unmatched individuals in the clinical surgical registry were more likely to be those who did not have coverage under Medicare, rather than individual whose correct match could not be found [25]. Consequently, differences in matched and unmatched individuals likely reflect the demographics of those who are/are not covered under Medicare. Similarly, in a linkage of a survey cohort to a maternity registry, unmatched records were typically the result of individuals moving outside of the maternity registry catchment area, rather than the result of errors in the linkage process [22]. As such, any found bias is more likely to reflect the different demographics of individuals who emigrate, rather than reflect linkage quality issues.

In this paper, we attempt to address these methodological shortcomings through the use of an alternate research design. Four large, real-world Australian administrative datasets were de-duplicated, with results and compared to an available ‘truth-set’, allowing comparison of both false positive and false negative errors.

## Methods

### Datasets

Each of the four separate administrative health datasets used in the evaluation contained multiple event-based records per person. The datasets comprised: ten years of Western Australian (WA) hospital admission data (*n* = 6,772,949), ten years of New South Wales (NSW) hospital admissions data (*n* = 19,874,083), three years of NSW public emergency department (ED) presentation data (*n* = 4,304,459) and three years South Australian (SA) ED presentation data (*n* = 813,839). Each dataset contained errors typical of administrative data, including missing and incorrect identifiers, and identifiers that change over time. Each dataset had previously been de-duplicated (identifying the records within each dataset belonging to the same individual) to a high quality by jurisdictional linkage units (the Centre for Health Record Linkage, the Western Australian Data Linkage Branch, and SANT Data Link for NSW, WA, and SA datasets respectively). These linkage units utilised a variety of deduplication methods including probabilistic record linkage, intensive manual review of created links and quality assurance procedures to analyse and review potential errors [28, 29]. The links created by these linkage units have been further validated through their regular use in academic and government research [2]. The linkage units provided the present study with the results of their matching processes, allowing us to use this as a ‘gold-standard’ with which to compare the results of our deduplication of these datasets. The data was made available as part of proof of concept work for the Population Health Research Network [30].

### Record linkage methods

Each dataset was de-duplicated using a single probabilistic linkage strategy, based on a previously published ‘default’ linkage strategy [31] (no linkages were conducted between any of the four datasets). This default strategy utilised two sets of blocks (Soundex of surname concatenated with first initial, and full date of birth), with all available variables used in comparisons. String similarity measures (Jaro-Winkler metric) were used for alphabetic variables, while exact matching strategies were used for other variables. Agreement and disagreement weights were calculated from the available gold standard benchmark.

### Linkage quality metrics

Linkage error occurs when pairs of records are not complete or include wrong matches. Where record pairs are incorrectly assigned as belonging to the same individual, we have false positives. In situations where record pairs are incorrectly identified as belonging to different people, there are false negatives. The aim in linkage is to maximise the number of true positives and true negatives.

Linkage quality was evaluated using pair-based quality metrics. Precision and recall were calculated by comparing the found results to those in the gold-standard benchmark. Precision referred to the proportion of found record-pairs that were correct, while recall referred to the proportion of all correct record-pairs found. The calculations for the measures are shown below.

$$ Precision=\frac{TP}{TP+ FP},\kern0.5em Recall=\frac{TP}{TP+ FN}, $$

where

*TP =*: *number of true positives,*
*FP =*: *number of false positives, and*
*FN =*: *number of false negatives*

A linkage with a high precision will have few false positives; similarly, a linkage with high recall will have few false negatives. The arithmetic mean of precision and recall was taken to determine the appropriate threshold score for which to report results.

### Sociodemographic variables

We investigated differences in linkage quality within sub-groups of the population based on selected sociodemographic attributes: gender, age (via year of birth), geographical region and socioeconomic status. Year of birth was split into three categories; those born prior to 1950, those born from 1950 to 1979, and those born from 1980 onwards (roughly corresponding to those aged under 20–30, those aged 20–30 to 50–60 and those older than 50–60). Indices of geographic remoteness (Accessibility Remoteness Index of Australia: ARIA+ [32]) were used, derived from the individual’s residential address. This metric is defined by the distance required by persons to travel to access particular services. Geographical remoteness was classified into major cities, regional (semi-rural) areas, and remote areas. The SEIFA metric of social disadvantage was used; this was also derived using the individual’s residential address. The SEIFA score is developed using factor analysis of responses to the Australian census and includes information on income, education, employment, occupation and housing. The score is computed for small geographic areas (approximately 200 dwellings) [33]. The SEIFA score was classified into quintiles.

### Measuring extent of linkage bias

A de-duplication linkage was conducted on each of the four datasets, using the record linkage method described above, resulting in a series of matched record-pairs. Linkage quality was calculated for each of the sociodemographic characteristics by ignoring those record-pairs that did not include at least one record with that sociodemographic category. For example, linkage quality for individuals for remote areas was calculated by evaluating the linkage quality on all pairs of records of which at least one record was from a remote area. Pair-based linkage quality was calculated directly on these record-pairs. Additionally, the metrics were calculated for a range of threshold scores to investigate how linkage bias may change as the threshold is either increased or decreased.

As clinical content data for each dataset was not available, statistics on the incidence of hospital encounters (admissions per person-year; a crude indicator of health) were calculated for each socio-demographic- category, with results compared against results from the gold-standard benchmark.

## Results

The number of records within each sociodemographic category for each dataset is shown in Table 1. The proportion of records in each category was similar but not identical across datasets. Both ED datasets had lower proportions of older individuals, and higher proportions of persons born 1950–1979, compared with the hospital datasets. There were some differences between states in terms of remoteness, with Western Australia having a much larger proportion of individuals living in remote areas (7%) compared with the other states (0–1%), and New South Wales having a higher proportion of individuals living in regional areas. All datasets showed similar distributions for socioeconomic status, with a slightly higher proportion in the most disadvantaged quintile compared with the least disadvantaged. Additional file 1: Table S1 in the supplementary material describes the proportion of missing values in each of these datasets, stratified by sociodemographic category. All of the datasets had relatively low levels of missing values, with the exception of the NSW hospital dataset, for which roughly one third of name information was missing. Differences in the proportion of missing values could be found for some sociodemographic categories in particular datasets.

**Table 1:** Sociodemographic profile of each dataset - number and proportion of records in each sociodemographic category

	NSW Emergency	NSW Hospital	SA Emergency	WA Hospital
Total	4,304,459 (100%)	19,874,083 (100%)	813,839 (100%)	6,772,949 (100%)
*Remoteness*				
Major Cities	2,868,504 (67%)	14,351,493 (72%)	690,399 (85%)	5,045,362 (74%)
Regional	1,367,635 (32%)	5,263,797 (26%)	54,665 (7%)	1,199,149 (18%)
Remote	12,250 (0%)	137,540 (1%)	6333 (1%)	498,850 (7%)
*Sex*				
Male	2,178,168 (51%)	9,346,451 (47%)	386,176 (47%)	3,184,925 (47%)
Female	2,125,422 (49%)	10,526,591 (53%)	427,645 (53%)	3,588,021 (53%)
*Socioeconomic Status*				
Most Disadvantaged	1,155,081 (27%)	4,133,693 (24%)	212,613 (26%)	1,723,482 (26%)
2	833,405 (19%)	3,574,267 (21%)	172,838 (21%)	1,468,495 (22%)
3	974,884 (23%)	3,220,110 (19%)	154,871 (19%)	1,256,879 (19%)
4	705,006 (16%)	3,133,389 (18%)	129,227 (16%)	1,128,067 (17%)
Least Disadvantaged	563,326 (13%)	3,285,911 (19%)	103,915 (13%)	1,149,271 (17%)
*Year of birth*				
< 1950	1,431,527 (33%)	9,726,134 (49%)	269,257 (33%)	3,164,258 (47%)
1950–1979	1,849,952 (43%)	6,460,872 (33%)	353,815 (43%)	2,535,776 (37%)
1980+	1,022,980 (24%)	3,686,298 (19%)	190,767 (23%)	1,072,915 (16%)

Overall, the linkage quality achieved in the deduplication of each dataset was very high. Linkage results at the optimal threshold (the threshold that maximised the average of precision and recall scores) are shown in Table 2 below.

**Table 2:** Optimal overall linkage quality for each of the four datasets

	Precision	Recall	Average
NSW Emergency	0.993	0.988	0.991
NSW Hospital	0.986	0.971	0.979
SA Emergency	0.988	0.971	0.980
WA Hospital	0.994	0.987	0.991

**Fig. 1:**
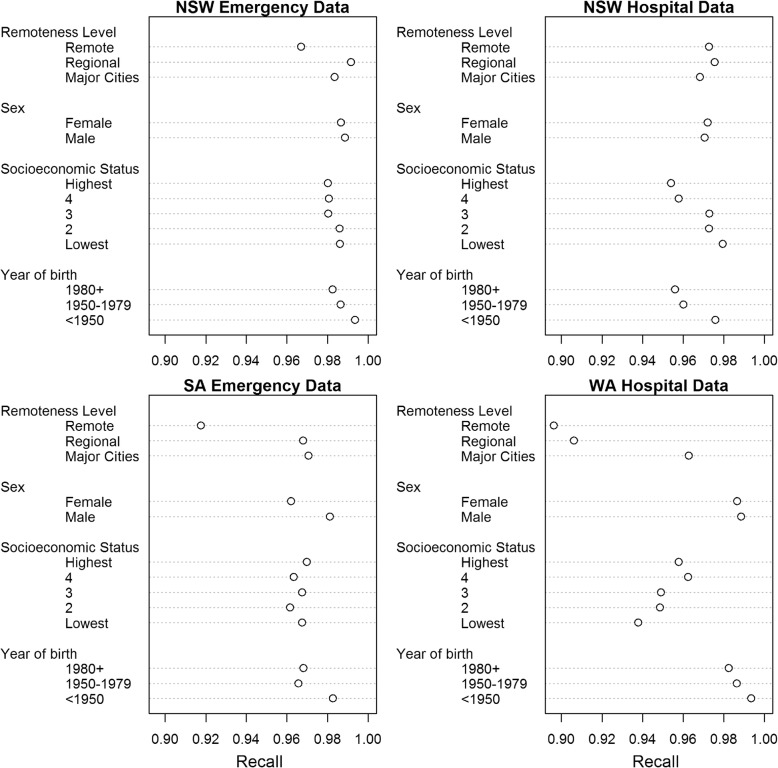
Comparison of recall scores at optimal threshold by sociodemographic category and dataset

**Fig. 2:**
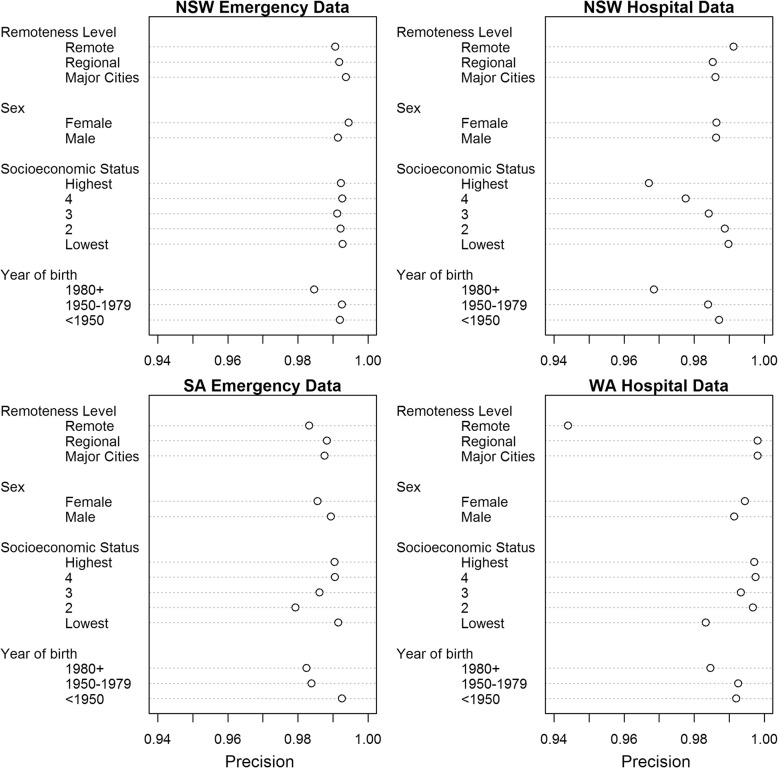
Comparison of precision scores at optimal threshold by sociodemographic category and dataset

Variations in linkage quality by sociodemographic category for each of the four datasets, calculated at the optimal threshold score, are shown in Figs. 1 and 2. Results varied between datasets, and by sociodemographic category. There was generally little difference in precision and recall between males and females, although there was a greater number of missed matches (lower recall) for females in SA emergency data (recall _male_ = 0.981, recall _female_ = 0.962). There was an identifiable effect of age on linkage quality, with persons born after 1980 having a lower linkage quality (both precision and recall) for all four datasets, as compared to those born before 1950 (on average a reduction of 0.01 for both precision and recall). The effect of geographic remoteness on linkage quality was not as clear. In the NSW emergency, SA emergency and WA hospital datasets, records of persons in remote areas had lower recall compared with those in major cities (NSW emergency; recall _major cities_: 0.984, recall _remote areas_: 0.967; SA emergency; recall _major cities_: 0.971, recall _remote areas_: 0.918; WA hospital recall _major cities_: 0.963, recall _remote areas_: 0.896 . The proportion of false matches (precision) was only noticeably greater for those in remote areas in the WA hospital dataset (precision = 0.944 compared to 0.998 for both regional areas and major cities). For socioeconomic status, the effect on linkage quality, in terms of precision and recall, was even less clear. In the WA hospital dataset, precision and recall both generally decreased as socioeconomic status decreased, with those in the upper quintiles having the highest linkage quality. For the NSW hospital dataset, however, the effect was the opposite, with precision and recall generally decreasing as socioeconomic status increased. Results for the NSW emergency dataset showed little difference in precision and recall between socioeconomic quintiles, while the results for SA emergency data did not show any clear trend.

We further determined the optimal threshold score for each particular sociodemographic factor (i.e. calculating precision and recall using separate thresholds for those in major cities, regional and remote areas). However, this resulted in no substantial change in linkage quality (changes were extremely small, beyond the fourth significant figure).

Comparisons of incidence rates for each dataset and sociodemographic category are shown in Table 3. Differences, where they existed, were generally small.

**Table 3:** Incidence rates (admissions/presentations per person-year) by sociodemographic category and dataset, comparing the gold-standard benchmark to the linkage results

	NSW Emergency		NSW Hospital		SA Emergency		WA Hospital	
	GS^a^	Estimated	GS	Estimated	GS	Estimated	GS	Estimated
*Remoteness*								
Major Cities	0.68	0.68	0.41	0.41	0.75	0.76	0.47	0.48
Regional	0.83	0.84	0.42	0.43	0.53	0.53	0.46	0.46
Remote	0.59	0.590	0.46	0.47	0.50	0.50	0.48	0.52
*Sex*								
Male	0.72	0.73	0.42	0.42	0.70	0.70	0.46	0.47
Female	0.72	0.72	0.41	0.41	0.75	0.75	0.47	0.47
*Socioeconomic Status*								
Most Disadvantaged	0.85	0.86	0.44	0.45	0.83	0.84	0.54	0.56
2	0.64	0.65	0.41	0.41	0.75	0.76	0.48	0.49
3	0.82	0.83	0.39	0.38	0.71	0.72	0.46	0.47
4	0.65	0.66	0.38	0.37	0.66	0.66	0.43	0.43
Least Disadvantaged	0.59	0.59	0.38	0.38	0.60	0.61	0.41	0.41
*Year of birth*								
< 1950	0.78	0.79	0.66	0.69	0.76	0.76	0.76	0.79
1950–1979	0.68	0.69	0.36	0.36	0.70	0.70	0.42	0.42
1980+	0.73	0.74	0.29	0.27	0.75	0.76	0.31	0.3

^a^Results using the gold standard benchmark

## Discussion

There was some evidence that linkage quality varied across and within different sociodemographic categories. While some differences were found for gender and socioeconomic status, these results occurred only in particular datasets. The finding of poorer linkage quality for those in remote areas was more consistent, appearing in three of the four datasets. The most reliable finding was decreased linkage quality for younger individuals as compared to older individuals, found in all four datasets.

There are a number of reasons why poorer linkage quality may be expected in younger individuals and individuals who live in more remote areas. Younger individuals are more mobile and so change address more often [34]. Birth records in particular will often be missing the infants first name and may be given the surname of the mother, making them particularly difficult to link. Those in remote areas are more likely to be Indigenous (28% Indigenous in remote areas, as compared to 1% in major cities). While data indicating the Indigenous status of individuals was not directly available in our datasets, previous studies have shown Indigenous Australians to be associated with poorer linkage quality [35]. Smaller remote hospitals may have fewer resources available to ensure high quality recording standards. Those in remote areas are also more likely to be transferred to larger hospitals in more populous areas, resulting in a second admission with patient details again recorded, providing greater opportunity for mistakes. While no high quality studies exist comparing levels of geographic remoteness, several previous studies have found lower linkage quality in younger individuals [36, 37].

The results found in this study suggests differences in linkage error across sociodemographic categories are highly dataset dependent. Specific quirks in recording standards in some data collections account for some of these findings. For instance, the NSW hospital admissions dataset did not contain name information for individuals attending private hospitals (containing only date of birth and address information). This is likely the reason for the finding in this dataset that linkage quality decreases as individuals have higher socioeconomic status, since those with higher socioeconomic status are more likely to attend private hospitals, and so have missing name data. It is quite possible that other findings in our dataset also reflect specific recording anomalies that we are not aware of.

The differences found in linkage quality were generally small, with a few larger differences (a drop in recall of around 0.05) found in remote areas. These differences in linkage quality had little notable effect on incidence rates. The effect of these errors on other derived clinical indicators is an important area of further study.

The use of subgroup-specific threshold settings made little difference to overall linkage quality, and based on these results cannot be recommended. Other published techniques such as the use of graph theory methods [38] or alternate grouping strategies [39] may be more fruitful methods of improving linkage quality across subpopulations. It remains to be proved whether other measures, such as the use of specific linkage strategies, parameters or techniques for specific subpopulations can lead to improvements in linkage quality for these groups.

A key strength of our study was the use of validated, real world datasets where the ‘answer’ in relation to linkage results is known. This allowed us to assess both false positive and false negative errors in record linkage. The use of a gold standard benchmark avoids the difficulty associated with determining whether non-matched records are the result of linkage error or some other reason (i.e. data capture error), a key weakness in many previously published studies. It should be noted that while the gold-standard results used in this study were of very high quality due to extensive clerical review and quality assurance, errors may still exist within the gold-standard results.

This study utilised a ‘default’ probabilistic linkage strategy, which we consider a relatively standard approach, and which achieves high quality results. As such, the level of error found in this study we consider to be small. While we could deliberately degrade the linkage strategy to reduce linkage quality and therefore amplify the level of bias in the results, we would risk introducing bias that would not typically be found in practice.

Information on additional subpopulations that may have higher risk of lower linkage quality, such as Indigenous Australians, immigrant groups, those with poorer health status, and those with mental health conditions, was not available but further research in these areas is warranted. Our study used structured and defined administrative datasets, with high quality data collection standards; as a result, overall linkage quality was very high. Data collections with overall poorer quality may produce magnified or alternate results to those found in this study. Interactions are likely to exist between several variables that influence linkage quality. For instance, Indigenous Australians make up a greater proportion of the population in remote areas as compared to major cities and are more likely to be from lower socioeconomic status areas. In addition, regional and especially remote areas generally have lower socioeconomic status as compared to major cities. Interaction effects such as these were not explored in this study. It is also not clear whether the number of existing records per person influences the difficulty of linking data related to that individual; this may interact with the tested sociodemographic categories, particularly those related to age.

## Conclusions

Our results suggest linkage quality in those younger and those living in remote areas may be lower, as a result, caution should be taken when analysing differences in linked Australian hospital data by age or geographic remoteness. Further research is required to determine the ramifications of these results for researchers utilising linked data.

## Additional file

Additional file 1: **Table S1.** Proportion of missing values in each dataset, stratified by sociodemographic variable. (DOCX 21 kb)

## Abbreviations

ED: Emergency department
NSW: New South Wales
SA: South Australia
WA: Western Australia
