# Supplementary material for: Sociodemographic differences in linkage error: an examination of four large-scale datasets
Source: BMC Health Serv Res. 2018 Sep 3;18:678. doi: 10.1186/s12913-018-3495-x (PMC6122711; doi:10.1186/s12913-018-3495-x)
Supplement: Supplementary file 1 — Table S1. Proportion of missing values in each dataset, stratified by sociodemographic variable. (DOCX 21 kb) [file 12913_2018_3495_MOESM1_ESM.docx]

Additional Material

Table S1: Proportion of missing values in each dataset, stratified by sociodemographic variable.

|  | **First Name** | | **Middle Name** | **Surname** | | **DOB** | **Sex** | | **Address** | **Suburb** | | **Postcode** |
| --- | --- | --- | --- | --- | --- | --- | --- | --- | --- | --- | --- | --- |
| **NSW Emergency** | | |  |  | |  |  | |  |  | |  |
| Total | 0.2% | | 83.4% | 0.0% | | 0.0% | 0.0% | | 4.2% | 1.3% | | 1.2% |
| *Remoteness* |  | |  |  | |  |  | |  |  | |  |
| Major Cities | 0.2% | | 89.0% | 0.0% | | 0.0% | 0.0% | | 0.4% | 0.1% | | 0.0% |
| Regional | 0.0% | | 71.6% | 0.0% | | 0.0% | 0.0% | | 10.3% | 0.3% | | 0.0% |
| Remote | 0.0% | | 73.9% | 0.0% | | 0.0% | 0.0% | | 3.0% | 0.0% | | 0.0% |
| *Sex* |  | |  |  | |  |  | |  |  | |  |
| Male | 0.1% | | 83.1% | 0.1% | | 0.0% | 0.0% | | 4.3% | 1.5% | | 1.5% |
| Female | 0.2% | | 83.8% | 0.0% | | 0.0% | 0.0% | | 4.1% | 1.1% | | 1.0% |
| *Socioeconomic Status* | |  | | |  | | |  | | |  | |
| Most Disadvantaged | 0.3% | | 80.6% | 0.0% | | 0.0% | 0.0% | | 5.2% | 0.3% | | 0.1% |
| 2 | 0.2% | | 95.2% | 0.0% | | 0.0% | 0.0% | | 8.2% | 0.1% | | 0.1% |
| 3 | 0.1% | | 71.7% | 0.0% | | 0.0% | 0.0% | | 1.6% | 0.1% | | 0.1% |
| 4 | 0.1% | | 85.8% | 0.0% | | 0.0% | 0.0% | | 0.8% | 0.1% | | 0.1% |
| Least Disadvantaged | 0.0% | | 88.9% | 0.0% | | 0.0% | 0.0% | | 0.7% | 0.4% | | 0.1% |
| *Year of birth* |  | |  |  | |  |  | |  |  | |  |
| <1950 | 0.2% | | 83.9% | 0.0% | | 0.0% | 0.0% | | 3.6% | 0.6% | | 0.5% |
| 1950-1979 | 0.2% | | 83.4% | 0.0% | | 0.0% | 0.0% | | 4.2% | 1.2% | | 1.2% |
| 1980+ | 0.1% | | 82.9% | 0.0% | | 0.0% | 0.0% | | 5.0% | 2.3% | | 2.4% |
| **NSW Hospital** | | |  |  | |  |  | |  |  | |  |
| Total | 33.9% | | 68.8% | 31.9% | | 0.0% | 0.0% | | 7.5% | 0.3% | | 0.6% |
| *Remoteness* |  | |  |  | |  |  | |  |  | |  |
| Major Cities | 36.2% | | 75.1% | 34.1% | | 0.0% | 0.0% | | 7.0% | 0.1% | | 0.0% |
| Regional | 28.3% | | 51.9% | 26.7% | | 0.0% | 0.0% | | 8.2% | 0.1% | | 0.0% |
| Remote | 15.9% | | 60.2% | 13.9% | | 0.0% | 0.0% | | 5.0% | 0.0% | | 0.0% |
| *Sex* |  | |  |  | |  |  | |  |  | |  |
| Male | 32.7% | | 68.2% | 30.6% | | 0.0% | 0.0% | | 7.0% | 0.3% | | 0.7% |
| Female | 34.9% | | 69.4% | 33.0% | | 0.0% | 0.0% | | 8.0% | 0.3% | | 0.5% |
| *Socioeconomic Status* | |  | | |  | | |  | | |  | |
| Most Disadvantaged | 17.9% | | 58.1% | 15.2% | | 0.0% | 0.0% | | 0.0% | 0.0% | | 0.1% |
| 2 | 24.6% | | 62.3% | 22.4% | | 0.0% | 0.0% | | 0.0% | 0.1% | | 0.1% |
| 3 | 29.7% | | 68.5% | 27.6% | | 0.0% | 0.0% | | 0.0% | 0.0% | | 0.1% |
| 4 | 36.0% | | 72.2% | 33.9% | | 0.0% | 0.0% | | 0.0% | 0.0% | | 0.0% |
| Least Disadvantaged | 43.1% | | 76.6% | 41.5% | | 0.0% | 0.0% | | 0.0% | 0.0% | | 0.0% |
| *Year of birth* |  | |  |  | |  |  | |  |  | |  |
| <1950 | 33.0% | | 67.8% | 32.9% | | 0.0% | 0.0% | | 7.0% | 0.3% | | 0.4% |
| 1950-1979 | 35.6% | | 69.8% | 35.3% | | 0.0% | 0.0% | | 8.7% | 0.2% | | 0.8% |
| 1980+ | 33.1% | | 70.0% | 23.4% | | 0.0% | 0.0% | | 7.1% | 0.2% | | 0.7% |
| **SA Emergency** | | |  |  | |  |  | |  |  | |  |
| Total | 2.2% | | 74.4% | 1.3% | | 0.0% | 0.0% | | 4.6% | 3.3% | | 7.5% |
| *Remoteness* |  | |  |  | |  |  | |  |  | |  |
| Major Cities | 0.9% | | 74.2% | 0.0% | | 0.0% | 0.0% | | 0.9% | 0.6% | | 0.0% |
| Regional | 0.5% | | 71.5% | 0.0% | | 0.0% | 0.0% | | 15.1% | 0.1% | | 0.0% |
| Remote | 0.6% | | 74.3% | 0.0% | | 0.0% | 0.0% | | 23.7% | 0.5% | | 0.0% |
| *Sex* |  | |  |  | |  |  | |  |  | |  |
| Male | 2.1% | | 73.3% | 1.4% | | 0.0% | 0.0% | | 4.6% | 3.4% | | 7.9% |
| Female | 2.3% | | 75.3% | 1.3% | | 0.0% | 0.0% | | 4.5% | 3.2% | | 7.1% |
| *Socioeconomic Status* | |  | | |  | | |  | | |  | |
| Most Disadvantaged | 1.0% | | 74.3% | 0.0% | | 0.0% | 0.0% | | 1.4% | 0.8% | | 3.2% |
| 2 | 1.0% | | 74.1% | 0.0% | | 0.0% | 0.0% | | 1.7% | 0.6% | | 4.8% |
| 3 | 0.8% | | 74.1% | 0.0% | | 0.0% | 0.0% | | 1.7% | 0.5% | | 3.3% |
| 4 | 0.8% | | 74.1% | 0.0% | | 0.0% | 0.0% | | 2.8% | 0.2% | | 1.5% |
| Least Disadvantaged | 0.7% | | 73.0% | 0.0% | | 0.0% | 0.0% | | 3.4% | 0.9% | | 2.1% |
| *Year of birth* |  | |  |  | |  |  | |  |  | |  |
| <1950 | 2.3% | | 76.3% | 1.5% | | 0.0% | 0.0% | | 4.8% | 3.5% | | 6.7% |
| 1950-1979 | 2.2% | | 74.4% | 1.4% | | 0.0% | 0.0% | | 4.8% | 3.4% | | 7.8% |
| 1980+ | 2.0% | | 71.7% | 1.1% | | 0.0% | 0.0% | | 3.7% | 2.8% | | 7.9% |
| **WA Hospital** | | |  |  | |  |  | |  |  | |  |
| Total | 0.3% | | 40.8% | 0.0% | | 0.0% | 0.0% | | 0.2% | 0.1% | | 0.2% |
| *Remoteness* |  | |  |  | |  |  | |  |  | |  |
| Major Cities | 0.3% | | 40.3% | 0.0% | | 0.0% | 0.0% | | 0.0% | 0.0% | | 0.0% |
| Regional | 0.2% | | 33.1% | 0.0% | | 0.0% | 0.0% | | 0.1% | 0.0% | | 0.0% |
| Remote | 0.2% | | 64.0% | 0.0% | | 0.0% | 0.0% | | 0.3% | 0.0% | | 0.0% |
| *Sex* |  | |  |  | |  |  | |  |  | |  |
| Male | 0.3% | | 40.0% | 0.0% | | 0.0% | 0.0% | | 0.2% | 0.1% | | 0.2% |
| Female | 0.3% | | 41.6% | 0.0% | | 0.0% | 0.0% | | 0.1% | 0.0% | | 0.2% |
| *Socioeconomic Status* | |  | | |  | | |  | | |  | |
| Most Disadvantaged | 0.3% | | 42.2% | 0.0% | | 0.0% | 0.0% | | 0.1% | 0.0% | | 0.0% |
| 2 | 0.4% | | 40.3% | 0.0% | | 0.0% | 0.0% | | 0.1% | 0.0% | | 0.0% |
| 3 | 0.3% | | 40.7% | 0.0% | | 0.0% | 0.0% | | 0.0% | 0.0% | | 0.0% |
| 4 | 0.3% | | 40.4% | 0.0% | | 0.0% | 0.0% | | 0.2% | 0.0% | | 0.0% |
| Least Disadvantaged | 0.3% | | 39.7% | 0.0% | | 0.0% | 0.0% | | 0.0% | 0.0% | | 0.0% |
| *Year of birth* |  | |  |  | |  |  | |  |  | |  |
| <1950 | 0.1% | | 42.5% | 0.0% | | 0.0% | 0.0% | | 0.0% | 0.0% | | 0.1% |
| 1950-1979 | 0.1% | | 40.2% | 0.0% | | 0.0% | 0.0% | | 0.3% | 0.2% | | 0.2% |
| 1980+ | 1.4% | | 37.3% | 0.0% | | 0.0% | 0.0% | | 0.2% | 0.1% | | 0.3% |
